# Supplementary material for: NMR-Based Metabolomic Analysis for the Effects of Trimethylamine N-Oxide Treatment on C2C12 Myoblasts under Oxidative Stress
Source: Biomolecules. 2022 Sep 13;12(9):1288. doi: 10.3390/biom12091288 (PMC9496509; doi:10.3390/biom12091288)
Supplement: Supplementary file 1 [file biomolecules-12-01288-s001.zip › biomolecules-1855461-supplementary.pdf]

Supplementary Materials

Figure S1.

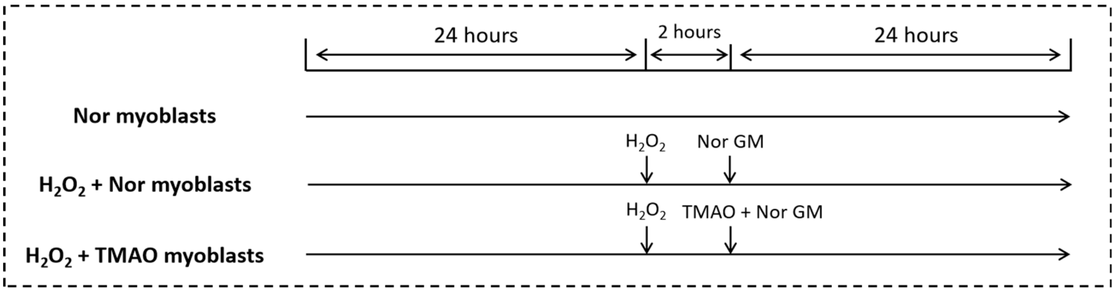

Figure S1. Schematic representation of the cell experimental design. C2C12 cells were firstly cultured in normal growth medium (Nor GM) to reach 50-60% confluence for 24 h. H_2O_2 was added to C2C12 myoblasts at a final concentration of 0.5 mM for 2 h, and then the medium was removed. Nor GM was added in the H_2O_2-treated (H_2O_2) group; TMAO was dissolved in normal growth medium to C2C12 myoblasts for 24 h in the TMAO-treated (TMAO) groups with three final concentrations of TMAO (1, 5 and 10 mM).

Figure S2.

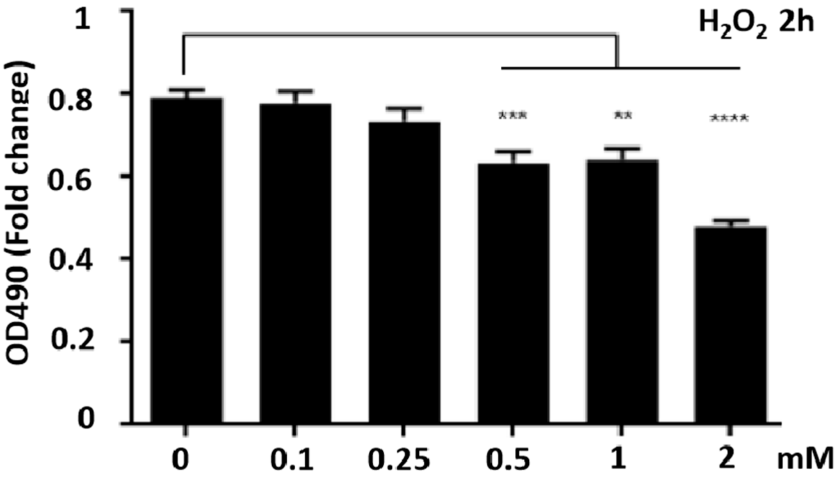

Figure S2. Cell viabilities of C2C12 myoblasts treated by H_2O_2 at various concentrations for 2h. Cell viabilities were assessed by measured $OD_{490\ nm}$ values with the MTS approach (n = 6). ** $p < 0.01$, ***$P < 0.001$, **** $p < 0.0001$

Figure S3.

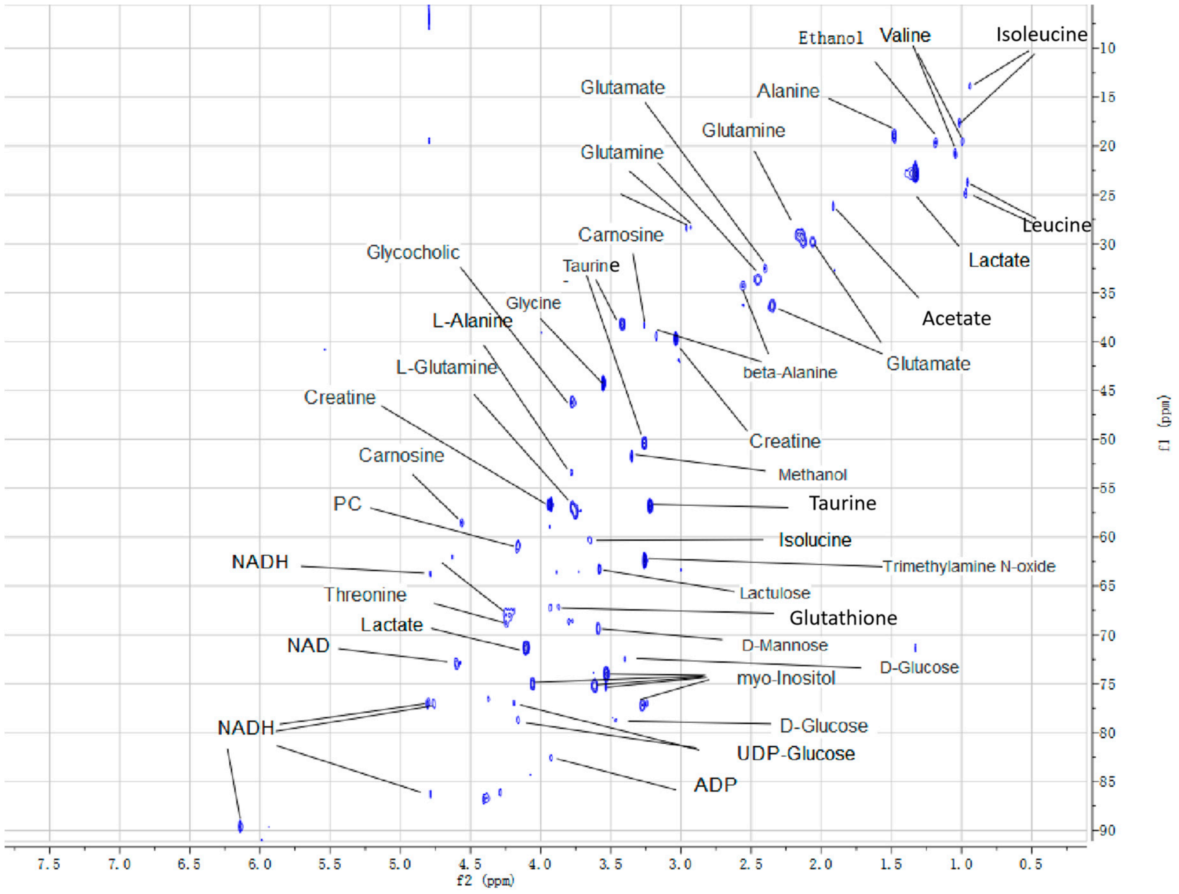

Figure S3. Representative 2D ^{1}H-^{13}C HSQC spectrum of aqueous extracts derived from C2C12 myoblasts recorded on 850 MHz NMR spectrometer.

Figure S4.

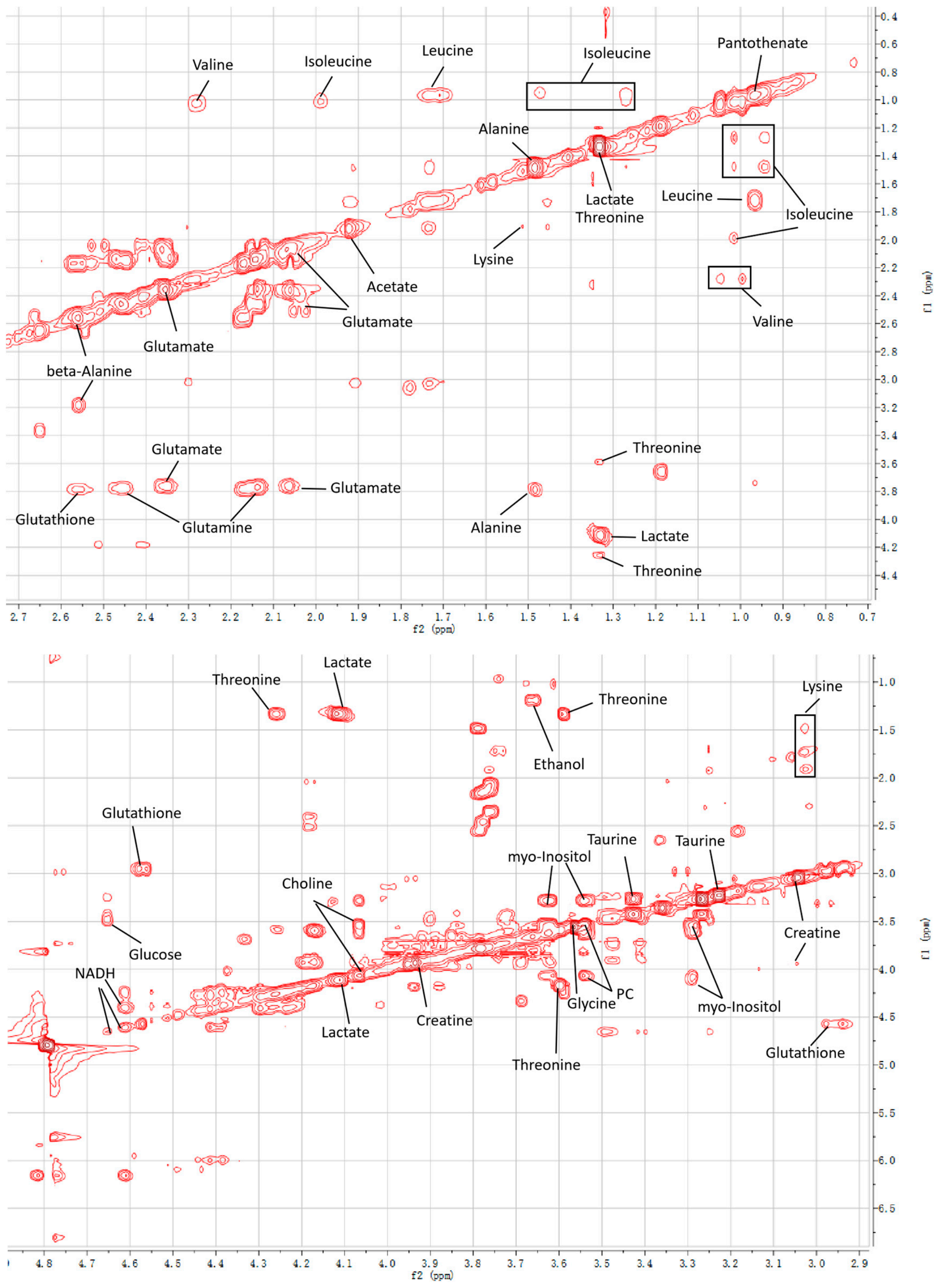
Valine
Isoleucine
Leucine
Isoleucine
Pantothenate
Alanine
Lactate
Threonine
Leucine
Isoleucine
Lysine
Acetate
Glutamate
Valine
Glutamate
beta-Alanine
Glutamate
Threonine
Glutamate
Glutathione
Glutamine
Alanine
Lactate
Threonine
f2 (ppm)
f1 (ppm)
Threonine
Lactate
Threonine
Lysine
Ethanol
Glutathione
Taurine
Taurine
myo-Inositol
Choline
Creatine
Glucose
NADH
PC
Glycine
myo-Inositol
Lactate
Creatine
Threonine
Glutathione
f2 (ppm)
f1 (ppm)

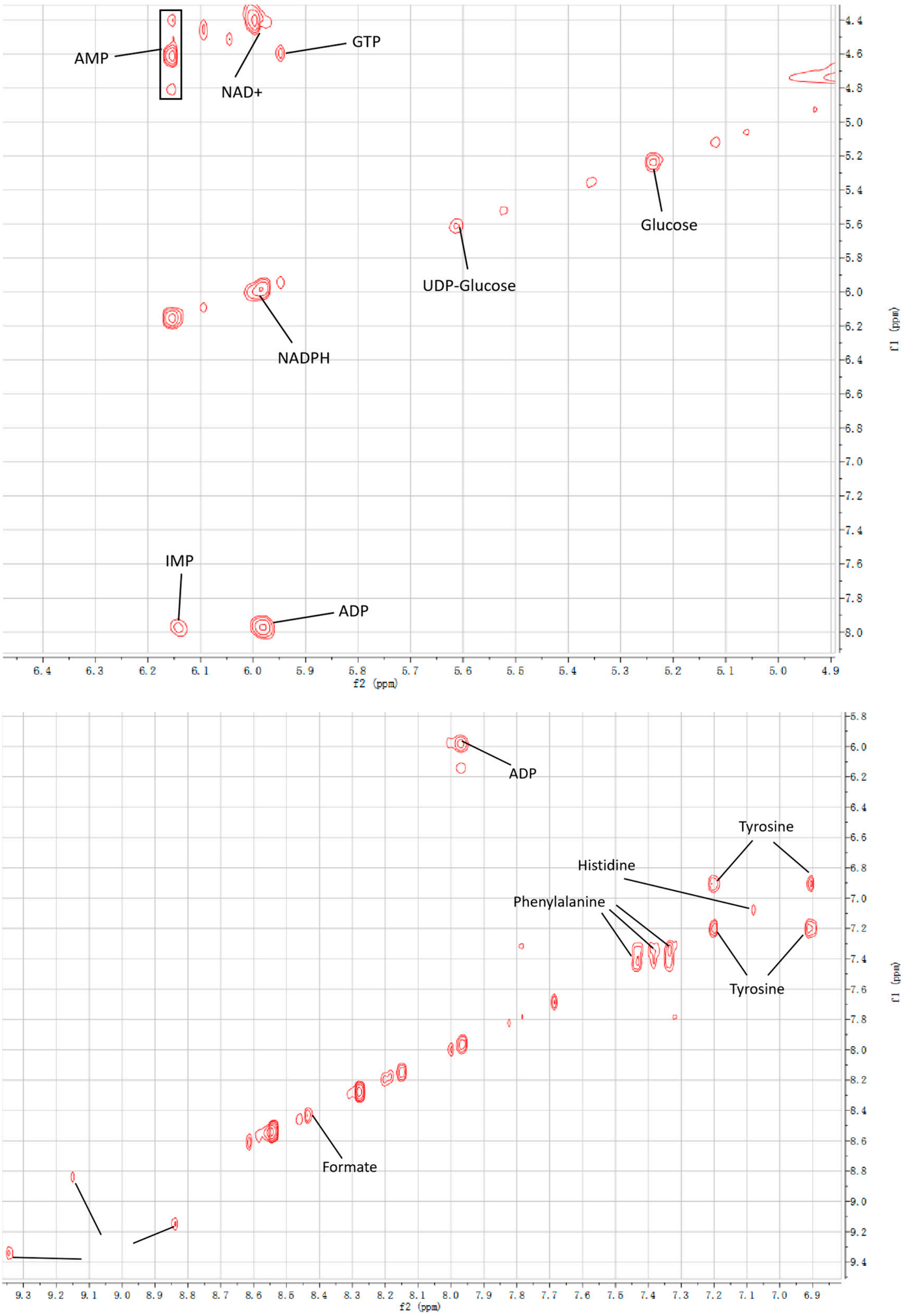

Figure S4. Representative 2D ^{1}H-^{1}H TOCSY spectrum of aqueous extracts derived from C2C12 myoblasts recorded on 850 MHz NMR spectrometer.

Figure S5.

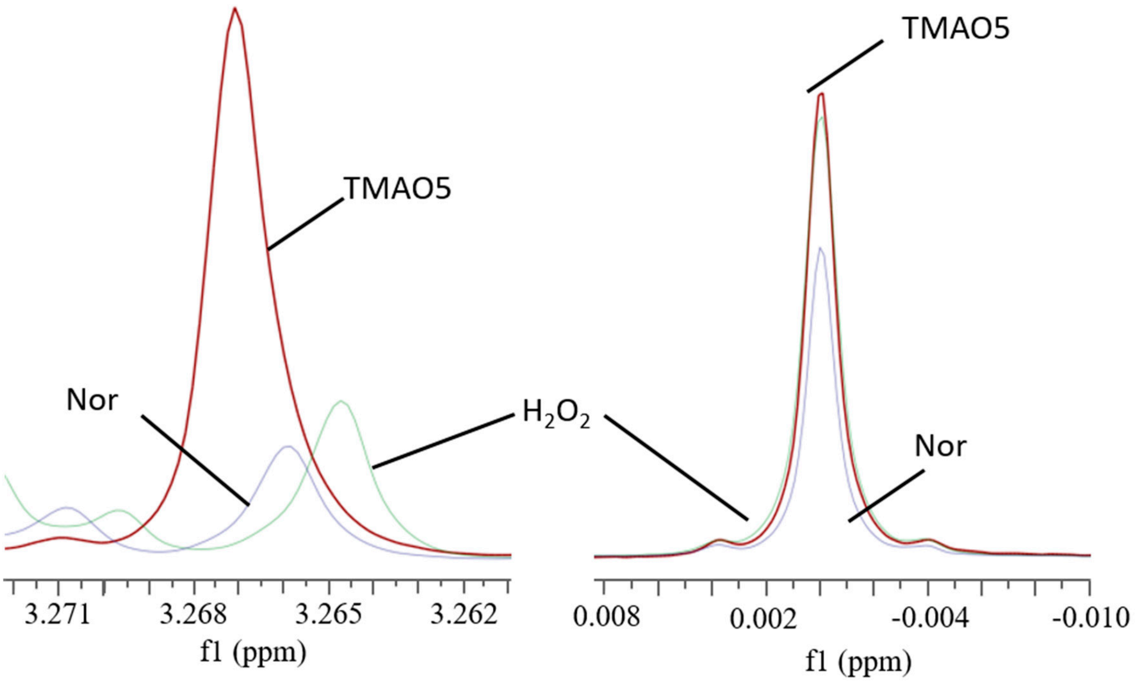

Figure S5. Local amplified regions of TMAO peaks in a typical 850 MHz ^{1}H NMR spectrum recorded on aqueous extracts derived from three groups of C2C12 myoblasts. The TSP peak was used as a chemical shift reference (δ 0.00). Blue line: spectral region from the Nor group. Green line: spectral region from the H_2O_2 group. Brown line: spectral region from the TMAO5 group.

Table S1. Identified metabolites in ^{1}H NMR spectra of aqueous extracts derived from C2C12 myoblasts

NO.	Metabolites	δ ^{1}H (ppm) and multiplicity	Moieties
1	Pantothenate	0.90(s), 0.94(s)	CH_3, CH_3
2	Isoleucine	0.93(t), 1.00(d), 1.26(m), 1.42(m), 1.99(m), 3.66(d)	δ-CH_3, γ-CH_3, half γ-CH_2, half γ-CH_2, β-CH, α-CH
3	Leucine	0.94(d), 0.97(d), 1.71(m), 1.74(m), 3.73(m)	α-CH_3, α-CH_3, γ-CH, β-CH_2, α-CH
4	Valine	0.99(d), 1.05(d), 2.26(m), 3.60(d)	γ-CH_3, γ-CH_3, β-CH, α-CH
5	Ethanol	1.17 (t), 3.65 (q)	β-CH_3, CH_2
6	Alanine	1.47(d), 3.78(q)	β-CH_3, α-CH
7	Putrescine	1.76 (m), 3.04 (m)	2CH_2, 2N–CH_2
8	Acetate	1.91(s)	CH_3
9	Glutamate	2.08(m), 2.12(m), 2.34(m), 2.37(m), 3.75(m)	half β-CH_2, half β-CH_2, half γ-CH_2, half γ-CH_2, α-CH
10	Glutamine	2.13(m), 2.42(m), 2.45(m), 3.77(t)	γ-CH_2, β-CH_2, α-CH
11	Methylamine (MA)	2.59 (s)	N–CH_3
12	Methionine	1.98 (m), 2.13 (s), 2.17 (m), 2.66 (dd), 3.78 (m)	δ-CH_3, γ-CH_2, β-CH_2
13	Dimethylamine (DMA)	2.72 (s)	CH_3
14	Sarcosine	2.74 (s), 3.6 (s)	N–CH_3, α-CH_2
15	Trimethylamine (TMA)	2.88 (s)	CH_3
16	Glutathione	2.15(m), 2.55(m), 2.96(m), 3.77(m), 4.56(m)	β-CH_2, γ-CH_2, CH_2-SH, α-CH&CH_2-NH, CH-NH
17	Lysine	1.43(m), 1.50(m), 1.73(m), 1.89(m), 1.92(m), 3.02(t), 3.75(t)	γ-CH_2, half γ-CH_2, δ-CH_2, β-CH_2, ε-CH_2, α-CH
18	Phosphocreatine (PCr)	3.05(s), 4.05(s)	N-CH_3, CH_2
19	beta-Alanine	2.54(t), 3.17(t)	CH_2, CH_2
20	Choline	3.21(s), 3.50(dd), 4.03(t)	N-$(CH_3)_3$, N-CH_2, CH_2OH
21	sn-Glycero-3-phosphocholine (GPC)	3.23(s), 3.60(dd), 3.68(dd), 3.87(m), 3.94(m), 4.33(m)	N-$(CH_3)_3$, half $^{1}CH_2$, $^{2}CH_2$, half $^{1}CH_2$, half $^{3}CH_2$, half $^{3}CH_2$, $^{1}CH_2$
22	Trimethylamine N-oxide (TMAO)	3.26(s)	CH_3
23	Taurine	3.24(t), 3.41(t)	$^{1}CH_2$, $^{2}CH_2$
24	myo-Inositol	3.28(t), 3.53(dd), 3.63(t), 4.07(t)	^{2}CH, $^{4,6}CH$, $^{1,3}CH$, ^{5}CH
25	Glycine	3.57(s)	α-CH_2
26	Threonine	1.31(d), 3.59(d), 4.25(m)	γ-CH_2, β-CH
27	Creatine	3.04(s), 3.93(s)	N-CH_3, α-CH_2
28	Lactate	1.33(d), 4.11(q)	β-CH_3, α-CH

29	O-Phosphocholine (PC)	3.20(s), 3.59(t), 4.18(m)	N-$(CH_3)_3$, N-CH_2, CH_2OH
30	Glucose	β(3.24 (dd), 3.48 (t), 3.90 (dd)), α(3.54 (dd), 3.71 (t), 3.72 (dd), 3.83 (m))	β(H_2, H_3, H_5), α(H_2, H_3, H_6)
31	UDP-Glucose (UDPG)	5.62 (dd), 6.0 (m)	CH, 2CH
32	UDP-N-Acetylglucosamine (UDP-GlcNAc)	2.07 (s), 5.5 (q), 6.0 (dd), 7.94 (d), 8.3 (d)	CH_3, CH, 2CH, CH, NH
33	NADH	4.37(m), 4.17(m), 4.69(t), 6.14(d), 8.45(s)	CH, CH, CH, NH2, CH
34	Carnosine	2.68(m), 3.02(q), 7.06(s)	CH_2, CH
35	Tyrosine	3.05(dd), 3.19(dd), 6.92(d), 7.19(d)	half β-CH_2, half β-CH_2, β-CH, α-CH
36	Phenylalanine	3.12(dd), 3.30(dd), 3.99(dd), 7.33(d), 7.37(t),7.43(t)	α-CH, half β- CH_2, half β-CH_2, α-CH, β-CH, γ-CH
37	Pyridoxine	2.46(s), 4.81(s), 7.68(s),	CH_3, CH_2, CH
38	Histidine	7.06(s), 7.85(s)	^{5}CH, ^{2}CH
39	GTP	5.92 (d), 8.1 (s)	CH, CH
40	AMP	6.14 (d), 8.27 (s), 8.58 (s)	NH_2, δ-CH, ^{2}CH
41	NAD+	6.03(d), 6.08(s), 8.16(s), 8.20(m), 8.41(s), 8.82(d), 9.13(d), 9.32(s)	NH_2, NH_2(CO), δ-CH, β-CH, ^{2}CH, γ-CH, α-CH
42	Formate	8.46(s)	CH
43	NADPH	4.20(m), 5.97(m), 8.23(s), 8.47(s)	CH, CH, CH, CH
44	ADP	6.13 (d), 8.27 (s), 8.58 (s)	NH_2, δ-CH, ^{2}CH
45	IMP	4.37(m), 6.14(d), 8.22 (s), 8.58 (s)	CH, NH_2, CH, CH

Multiplicity: s, singlet; d, double; t, triplet; q, quartet; m, multiple; dd, double of double. Abbreviations: DMA, dimethylamine; TMA, trimethylamine; PCr, phosphocreatine; GPC, sn-Glycero-3-phosphocholine; TMAO, trimethylamine N-oxide; PC, O-phosphocholine; UDPG, UDP-glucose; UDP-GlcNAc, UDP-N-acetylglucosamine; GTP, guanosine triphosphate; AMP, adenosine monophosphate; NAD+, nicotinamide adenine dinucleotide; ADP, adenosine diphosphate; IMP, hypoxanthine nucleotide.
